# Optimized Oral Glutamatergic Augmentation in Refractory Home-Dominant Contamination Obsessive-Compulsive Disorder With Somatic, Affective, and Motivational Features: A Case Report of Incremental Functional Gains

**DOI:** 10.7759/cureus.111573

**Published:** 2026-06-26

**Authors:** Hoi Ki Cheung, Ngo Cheung

**Affiliations:** 1 Clinical Assistance, Chinese University of Hong Kong, Hong Kong, HKG; 2 Psychiatry, Cheung Ngo Medical Limited, Hong Kong, HKG

**Keywords:** contamination ocd, cyp2d6 inhibition, dextromethorphan, glutamatergic augmentation, home-dominant rituals, l-glutamine, piracetam, somatic hypochondriacal features, synaptic plasticity, treatment-resistant obsessive-compulsive disorder

## Abstract

A proportion of patients with obsessive-compulsive disorder (OCD) continue to experience disabling symptoms despite standard serotonergic treatment, antipsychotic augmentation, and psychological input. Contamination-focused OCD can be especially persistent when rituals are embedded in the home environment and reinforced by family patterns. Recent work has drawn attention to glutamatergic dysfunction, NMDA-AMPA signaling, and synaptic plasticity as possible treatment targets in refractory OCD. The Cheung glutamatergic regimen has been proposed as an oral, low-cost, ketamine-informed glutamatergic strategy using dextromethorphan, CYP2D6 inhibition, piracetam, and L-glutamine to modulate glutamatergic plasticity. The supporting evidence for this proposed regimen remains preliminary, largely case-based, and not independently replicated. A 26-year-old woman presented with several years of contamination fears, repetitive handwashing, prolonged bathing lasting up to 1.5 hours, home-dominant cleaning concerns, anxiety, depressive symptoms, fatigue, somatic complaints, nightmares, and motivational impairment. Baseline screening showed a Patient Health Questionnaire-9 (PHQ-9) of 18 and a Generalized Anxiety Disorder-7 (GAD-7) of 14, with passive suicidal ideation endorsed on one item. Over approximately nine months, treatment was adjusted in routine outpatient care. A glutamatergic-oriented augmentation strategy was used as part of a complex, evolving regimen built around dextromethorphan, piracetam, and later L-glutamine, with pharmacokinetic potentiation via CYP2D6-inhibiting antidepressants, including fluoxetine, bupropion, and later low-dose paroxetine. Low-dose risperidone, clomipramine, pregabalin, propranolol, cyproheptadine, and other symptom-focused adjuncts were used and adjusted according to response and tolerability.

Mood, energy, somatic chest discomfort, motivation, and resilience improved. Bathing time decreased from approximately 1.5 hours to about 30 minutes, and ritual preoccupation was at one point described as 20-30% reduced. The PHQ-9 self-harm item changed from “several days” at the first standardized assessment to “not at all” at Day 41, although passive ideation was again endorsed for several days during later stress. She resumed job-seeking, attended interviews, exercised, learned new skills, and coped better than expected with the illness and death of a long-term pet. Residual contamination and cleaning symptoms remained most prominent at home. No Yale-Brown Obsessive Compulsive Scale, formal ritual count, or structured home-versus-outside severity scale was recorded.

This case is best read as a hypothesis-generating clinical observation rather than evidence of regimen-specific efficacy. The response was incomplete but clinically meaningful, especially in affective, somatic, motivational, and functional domains. Larger controlled studies are needed, and home-targeted exposure-based work remains essential.

## Introduction

Obsessive-compulsive disorder (OCD) is a common and often disabling condition marked by intrusive thoughts, repetitive behaviors, avoidance, and attempts to neutralize distress. Contamination fears and cleaning rituals are among the best-known presentations, but their severity varies widely. In some patients, the disorder is not confined to visible rituals. It may involve prolonged internal doubt, body checking, shame, family conflict, task avoidance, and exhaustion after repeated attempts to feel “clean enough.” Standard treatments include cognitive-behavioral therapy with exposure and response prevention and serotonin reuptake inhibitors, often at higher doses than those used for depression [1,2]. Meta-analytic evidence supports a dose-response relationship for selective serotonin reuptake inhibitors in OCD, although higher doses may increase side-effect burden [3]. For patients with persistent symptoms, antipsychotic augmentation is commonly used, but response is not guaranteed, and adverse effects can limit long-term use [4,5].

Refractory contamination-focused OCD can be particularly difficult when symptoms are tied to the patient’s home. The home is not merely a setting where symptoms occur; it may become the main trigger network. Family accommodation, repeated reassurance, criticism, modeling of cleaning behavior, and shared household routines may all maintain symptoms even when the patient functions better outside the home [6,7]. This context-specific pattern complicates treatment. A patient may look more functional in public than at home, while the most severe rituals continue privately.

A growing body of work points beyond serotonin alone. Glutamate abnormalities have been implicated in OCD, especially within cortico-striato-thalamo-cortical circuits involved in habit, threat monitoring, and behavioral control [8,9]. Ketamine research has also encouraged interest in NMDA receptor modulation, AMPA throughput, and downstream synaptic plasticity. A proof-of-concept trial found rapid anti-obsessional effects after ketamine in OCD, although ketamine’s cost, monitoring requirements, and dissociative effects limit its routine use [10]. Preclinical work suggests that NMDA antagonism may rapidly engage AMPA-dependent pathways, mTOR signaling, and synapse formation, providing a plausible mechanism for fast changes in mood and compulsive circuitry [11-13].

The Cheung glutamatergic regimen was proposed as an oral approach based on the hypothesis that existing agents might engage some ketamine-relevant glutamatergic and plasticity pathways, using dextromethorphan for NMDA/sigma-related activity, a CYP2D6 inhibitor to prolong dextromethorphan exposure, piracetam to support AMPA receptor activity, and L-glutamine to support glutamate-glutamine cycling [14]. Early case reports and case series have described improvement in treatment-resistant obsessive-compulsive symptoms, somatic and hypochondriacal obsessions, trauma-related presentations, and refractory depression using variations of this approach [15-18]. These reports remain preliminary; several are preprints, most are from the same authorial group, and independent replication has not yet been established. These reports provide a clinical rationale for careful, documented single-case observations, but they do not establish efficacy.

This manuscript presents a detailed case of a 26-year-old woman with refractory, home-dominant contamination-focused OCD with anxiety, depressive symptoms, somatic complaints, nightmares, fatigue, and motivational impairment. The aim is not to claim remission, establish efficacy, or assign causality to any single medication component, but to describe a real-world course in which a glutamatergic-focused regimen was personalized over time, resulting in partial improvement in obsessive-compulsive symptoms and broader functional gains.

## Case presentation

History and presentation

A 26-year-old woman presented to outpatient psychiatric care with a several-year history of obsessive-compulsive symptoms centered on contamination fears (Table 1). Her main rituals included repeated handwashing, persistent doubts that her hands remained dirty even after washing, prolonged bathing, and home-focused cleaning concerns. Bathing could take up to 1.5 hours because she felt she had not washed herself thoroughly. She described resistance toward bathing because the process was exhausting, yet she remained anxious if prevented from cleaning. She also reported cleaning rituals throughout the day and recurring worries about whether she had touched rubbish bins or contaminated objects.

**Table 1:** Chronological summary of clinical course, symptom severity, tolerability, and pharmacological management over approximately nine months (36 weeks) in a 26-year-old woman with OCD and comorbid depressive-anxiety symptoms Data are de-identified. Weeks and days are approximated from the clinical baseline visit. Wk 0 refers to the clinical baseline, not the first standardized assessment. Severity bands are provided where scales were recorded. Dashes indicate that standardized symptom scales were not administered at that visit. Active medication lists were reconstructed from routine clinical notes; where continuation or discontinuation was not explicit, uncertainty is stated rather than inferred. PHQ-9: Patient Health Questionnaire-9, GAD-7: Generalized Anxiety Disorder-7 scale, AM: morning, BD: twice daily, nocte: nightly, CR/XL: controlled/extended release, PRN: as needed

Visit/date	Day/week from clinical baseline	OCD symptoms/outcomes	Standardized symptom scores, when recorded	Functional changes/stressors	Adverse effects/tolerability	Medication changes at visit	Active regimen after visit, as documented
Sep 2025	Day 0/Wk 0	Contamination obsessions, repeated compulsive handwashing, prolonged bathing about 1.5 h, home-focused cleaning concerns	Not administered at clinical baseline	Family and occupational stressors; part-time work; desire for full-time employment	None recorded at baseline	Started fluoxetine 20 mg AM; alprazolam 0.25 mg PRN; pregabalin 25 mg nocte; dextromethorphan 30 mg nocte	Fluoxetine 20 mg AM; alprazolam 0.25 mg PRN; pregabalin 25 mg nocte; dextromethorphan 30 mg nocte
Oct 2025	Day 8/Wk 1	Persistent contamination and ritualistic concerns	-	Early titration review	Not recorded	Fluoxetine increased to 30 mg AM	Fluoxetine 30 mg AM; alprazolam PRN; pregabalin nocte and dextromethorphan nocte were part of the early regimen
Oct 2025	Day 16/Wk 2	Persistent ritualistic preoccupation and continued thinking around rituals	PHQ-9 18, moderately severe; GAD-7 14, moderate; PHQ-9 self-harm item: several days	Less tired, more relaxed, less chest dullness compared with first presentation	Fatigue, nausea, depersonalization/floating or lightheaded feeling	Fluoxetine increased to 40 mg AM; risperidone 0.5 mg nocte introduced; alprazolam PRN continued	Fluoxetine 40 mg AM; risperidone 0.5 mg nocte; alprazolam PRN; early adjuncts continued where documented
Oct 2025	Day 27/Wk 4	Fewer rituals “in mind”; obsessions reduced only about 20-30%; persistent hygiene-related checking and rubbish-related contamination worries	-	Mood more stable; less tension; less chest dullness	No major new adverse effect recorded	Risperidone increased to 1 mg nocte	Fluoxetine 40 mg AM; risperidone 1 mg nocte; alprazolam PRN; adjunctive regimen continued where documented
Nov 2025	Day 41/Wk 6	Bathing time slightly reduced; repeated handwashing persisted; symptoms noted to be worse at home than outside	PHQ-9 10, moderate; GAD-7 9, mild; PHQ-9 self-harm item: not at all	Chest dullness gone; motivation to pack belongings increased; appeared more relaxed	Mild fatigue only	Fluoxetine increased to 60 mg AM	Fluoxetine 60 mg AM; risperidone 1 mg nocte; alprazolam PRN; adjunctive regimen continued where documented
Nov 2025	Day 55/Wk 8	Mood stable but minimal further improvement in compulsions; cleaning rituals remained predominantly home-based	-	Ongoing occupational uncertainty	Gastrointestinal symptoms noted	Bupropion XL 150 mg AM added; dextromethorphan increased to 45 mg nocte; Buscopan briefly prescribed for gastrointestinal symptoms	Fluoxetine 60 mg AM; risperidone 1 mg nocte; bupropion XL 150 mg AM; dextromethorphan 45 mg nocte; alprazolam PRN; short-term antispasmodic
Dec 2025	Day 69/Wk 10	No clearly quantified OCD change at this visit	-	Regimen expansion/maintenance review	Not recorded	Bupropion XL increased to 300 mg AM; piracetam 600 mg AM and nocte added; dextromethorphan split to 30 mg AM and 45 mg nocte	Fluoxetine 60 mg AM; risperidone 1 mg nocte; bupropion XL 300 mg AM; dextromethorphan 30 mg AM and 45 mg nocte; piracetam 600 mg BD; alprazolam PRN
Dec 2025	Day 83/Wk 12	Frequent obsessions persisted; she reported a clearer mind when resisting them; hypochondriacal thoughts appeared; bathing time decreased to about 30 min; compulsive buying of dolls persisted	-	Mood not markedly low	Sweating	Fluoxetine reduced to 40 mg AM; grounding techniques taught	Fluoxetine 40 mg AM; risperidone 1 mg nocte; bupropion XL 300 mg AM; dextromethorphan split dosing; piracetam 600 mg BD
Jan 2026	Day 111/Wk 16	Obsessive-compulsive thoughts could be resisted for longer, but obsessions persisted	PHQ-9 11, moderate; GAD-7 10, moderate; PHQ-9 self-harm item: several days	Fewer low-mood episodes; coping with elderly dog’s illness better than expected	Fatigue, sweating, headache, nightmares, morning hand sweating	Propranolol 20 mg BD planned; fluoxetine reduced to 30 mg AM; risperidone increased to 1.5 mg nocte	Fluoxetine 30 mg AM; risperidone 1.5 mg nocte; bupropion XL 300 mg AM; dextromethorphan split dosing; piracetam 600 mg BD; propranolol 20 mg BD
Jan 2026	Day 125/Wk 18	Obsession frequency and OCD symptoms remained similar	-	Dog had died; attended funeral; emotional reaction better contained than expected	Hand sweating reduced overall; nightmares continued	Clomipramine 25 mg nocte added; fluoxetine reduced to 20 mg AM; senna prescribed; dextromethorphan documented as 45 mg BD	Fluoxetine 20 mg AM; clomipramine 25 mg nocte; bupropion XL 300 mg AM; dextromethorphan 45 mg BD; piracetam 600 mg BD; propranolol 20 mg BD; risperidone 1.5 mg nocte; senna
Feb 2026	Day 140/Wk 20-21	Persistent OCD symptoms; no new quantified ritual outcome	-	Regimen consolidation	Constipation risk monitored	Clomipramine increased to 50 mg nocte; fluoxetine reduced to 10 mg AM; citicoline 500 mg AM added	Fluoxetine 10 mg AM; clomipramine 50 mg nocte; citicoline 500 mg AM; bupropion XL 300 mg AM; dextromethorphan 45 mg BD; piracetam 600 mg BD; propranolol 20 mg BD; risperidone 1.5 mg nocte
Mar 2026	Day 169/Wk 24	Mood stable but continued anxiety about touching dirty objects; clinician encouraged resisting compulsions	-	No major new functional change recorded	Constipation after clomipramine; amenorrhoea after risperidone	Bisacodyl and sennosides added for constipation; regimen otherwise maintained	Clomipramine 50 mg nocte; bupropion XL 300 mg AM; dextromethorphan 45 mg BD; piracetam 600 mg BD; propranolol 20 mg BD; risperidone 1.5 mg nocte; laxatives
Apr 2026	Day 197/Wk 28	Residual contamination symptoms persisted; no new quantified OCD outcome	-	Dose adjustment for tolerability	Fatigue and menstrual adverse effects considered clinically relevant	Risperidone reduced to 0.5 mg nocte; L-glutamine 500 mg daily added	Bupropion XL 300 mg AM; dextromethorphan 45 mg BD; piracetam 600 mg BD; clomipramine 50 mg nocte; L-glutamine 500 mg daily; risperidone 0.5 mg nocte
May 2026	Day 225/Wk 32	Residual contamination symptoms persisted, especially at home	-	Completed two short promoter-work periods; attended marketing/public-relations interviews; exercised; studied color analysis and marketing materials	Less tired after risperidone reduction	Trihexyphenidyl 2 mg BD added; target symptom not specified in available notes	Bupropion XL 300 mg AM; dextromethorphan 45 mg BD; piracetam 600 mg BD; clomipramine 50 mg nocte; L-glutamine 500 mg daily; risperidone 0.5 mg nocte; trihexyphenidyl 2 mg BD
Jun 2026	Day 253/Wk 36	Residual OCD symptoms remained; fragmented sleep and low mood noted	-	Ongoing vocational efforts; no further standardized rating recorded	Menstruation more regular after risperidone reduction; bruising on and off; possible hemorrhoids; possible tablet malabsorption; dyspnoea/shortness-of-breath sensations; sweating	Clomipramine documented as “to reduce”; paroxetine CR 6.25 mg nocte added; pregabalin 50 mg nocte reintroduced; cyproheptadine 4 mg BD added for sweating	Bupropion XL 300 mg AM; dextromethorphan 45 mg BD; piracetam 600 mg BD; risperidone 0.5 mg nocte; L-glutamine 500 mg daily; paroxetine CR 6.25 mg nocte; pregabalin 50 mg nocte; cyproheptadine 4 mg BD; clomipramine final stop/continuation status was not explicit in the available medication list after the planned reduction

Her symptoms were accompanied by depressive and anxiety features, including low mood, fatigue, emotional lability, worthlessness, perceived invalidation by parents, and reduced motivation. Somatic symptoms included chest dullness, dizziness, sweating, and irritable bowel symptoms that worsened under stress. She recalled a period during full-time work when she needed to stay in the toilet for a long time because of bowel symptoms. She had previously undergone an ECG for chest symptoms, which was reportedly uneventful. She did not initially present with marked fear of serious illness, though hypochondriacal thoughts later emerged transiently.

Sleep was affected mainly by nightmares rather than prolonged sleep latency at the first visit. Some nightmares involved people who had traumatized or hurt her in the past, and she sometimes woke with shortness of breath. Pregabalin later appeared to reduce the emotional impact or recall of nightmares. She had previously received counseling and psychological input and had used hypnotics during periods of acute distress.

Functionally, she worked part-time as a clerk and interest-class teacher. She wanted full-time employment. Her last full-time job, in marketing, had ended approximately two years before the presentation. She remained able to go out and reported that her motivation was better outside the home. The mental status examination noted good self-care, makeup, artificial nails, neat dress, and crying spells when discussing parental criticism or invalidation.

Family context was clinically important. Her mother had long-standing obsessive cleaning traits, especially around floors; spent heavily on cleaning detergent; and hoarded cleaning products. Her father made critical comments about the patient’s employment and abilities, including remarks that she was underemployed or that her work did not match her degree. He was described as work-focused and possibly prone to panic symptoms. These family patterns were not treated as confirmed diagnoses in relatives. Still, they formed part of the formulation because the patient’s obsessive-compulsive symptoms were much worse at home than outside.

Baseline assessments

On Day 16, standardized screening showed a Patient Health Questionnaire-9 (PHQ-9) of 18 and a Generalized Anxiety Disorder-7 (GAD-7) of 14, indicating moderate to moderately severe depressive and anxiety symptoms. The PHQ-9 responses included anhedonia, low mood, sleep disturbance nearly every day, fatigue nearly every day, appetite change, self-critical thoughts, impaired concentration nearly every day, psychomotor change for several days, and passive thoughts that she would be better off dead or of self-harm for several days. The GAD-7 responses indicated frequent nervousness, difficulty controlling worry, excessive worry, difficulty relaxing, restlessness, irritability, and fear that something awful might happen. No prominent acute safety risk was recorded, and the PHQ-9 self-harm item was absent on Day 41 but reappeared for several days during later stress on Day 111.

Reported side effects at that stage included daily fatigue with substantial functional impact, intermittent nausea, and a floating or lightheaded feeling. At the same visit, she also reported feeling less tired and more relaxed than at the initial presentation, with reduced chest dullness. This suggests that early treatment had already produced some improvement, although side effects and residual obsessive thinking remained. No Yale-Brown Obsessive Compulsive Scale, formal insight scale, structured diagnostic interview, ritual count, contamination hierarchy, or home-versus-public severity scale was recorded at baseline or during follow-up.

Diagnostic reasoning and differential diagnosis

The working diagnosis was OCD with prominent contamination obsessions and washing/cleaning compulsions. This formulation was supported by fears of intrusive contamination, repetitive washing and cleaning performed to reduce distress, prolonged bathing, avoidance, functional impairment, and a marked home-dominant pattern. The diagnosis was clinical rather than based on a structured interview.

Several overlapping or alternative formulations were considered from the available clinical material. Illness anxiety disorder or somatic symptom disorder overlapped with dizziness, chest dullness, irritable bowel symptoms, sweating, and later transient hypochondriacal thoughts, but the dominant and most persistent syndrome remained contamination-focused OCD rather than illness fear alone. Depressive and generalized anxiety symptoms were clinically important and screen-positive, but they did not fully explain the specific ritualized washing, contamination doubt, and home-based cleaning pattern. Trauma-related symptoms were considered because nightmares sometimes involved people who had hurt or traumatized her, but the record did not document a full post-traumatic stress disorder syndrome.

Obsessive-compulsive personality traits were also considered, especially in the family context. Still, the patient’s own symptoms were experienced as distressing, exhausting, and functionally impairing rather than simply valued as orderliness or cleanliness. The mother’s cleaning and detergent-hoarding behaviors and the father’s critical or possibly panic-prone traits were treated as family-context observations, not as confirmed diagnoses in relatives. Compulsive buying, particularly the purchase of many dolls, was noted during follow-up. The available notes did not establish mania, hypomania, substance use, or an independent impulse-control disorder; it was therefore described cautiously as a compulsive or stress-related behavior requiring monitoring. Formal insight rating was not recorded, but the patient’s description of rituals as exhausting and her attempts to resist them suggested at least partial insight.

Treatment course

The following treatment course should be read as a sequence of overlapping medication changes in routine care, not as a controlled test of a single agent or a fixed protocol. The first documented pharmacological phase began on Day 1. Treatment included pregabalin 25 mg nightly, dextromethorphan 30 mg nightly, alprazolam 0.25 mg as needed, and fluoxetine 20 mg in the morning. This early combination targeted anxiety, nightmares, sleep-related distress, and obsessive-compulsive symptoms while introducing dextromethorphan as a glutamatergic component. Fluoxetine also served as a serotonergic agent and a CYP2D6 inhibitor, a relevant pharmacokinetic feature when used with dextromethorphan [14,19].

By Day 8, fluoxetine was increased by adding 10 mg to the existing 20 mg dose, while alprazolam and pregabalin were continued. On Day 16, after PHQ-9 18 and GAD-7 14 were recorded, the regimen was changed to fluoxetine 40 mg in the morning, risperidone 0.5 mg nightly, and alprazolam as needed. The patient reported less fatigue, greater relaxation, less chest dullness, and reduced nightmare impact with pregabalin. Still, she maintained her ritual pattern and kept thinking about them.

On Day 27, she reported fewer rituals “in mind,” less tiredness, a more stable mood, less fluctuation, less tension, and reduced chest dullness. Obsessive-compulsive symptoms were only slightly reduced, with estimates of around 20-30%. She remained worried about contamination, especially whether she had touched rubbish or dirty objects. Risperidone was increased to 1 mg nightly, while fluoxetine 40 mg and alprazolam as needed were continued.

On Day 41, PHQ-9 had improved to 10 and GAD-7 to 9. The item on suicidal ideation was now “not at all.” She reported that bathing time had slightly decreased, chest dullness had resolved, motivation to pack her things had increased, and she appeared more relaxed. However, repeated handwashing continued. A key observation emerged: obsessive-compulsive symptoms were more intense at home than in the street. Fluoxetine was increased to 60 mg in the morning; risperidone, 1 mg nightly, was continued; and alprazolam, as needed, remained available.

On Day 55, mood was stable, but obsessive-compulsive symptoms remained largely home-based and cleaning-centered, with little further improvement in core compulsions. Bupropion XL 150 mg in the morning was introduced; dextromethorphan was prescribed at 45 mg nightly; fluoxetine 60 mg was continued; and risperidone 1 mg was continued. Buscopan was also prescribed briefly for gastrointestinal symptoms. Bupropion may increase dextromethorphan exposure through CYP2D6 inhibition and has precedent in approved dextromethorphan-bupropion antidepressant treatment, but the combination requires attention to drug interactions and tolerability [20,21].

On Day 69, the glutamatergic strategy was expanded and split across the day. Piracetam, 600 mg in the morning and 600 mg nightly, was added. Dextromethorphan was prescribed as 30 mg in the morning and 45 mg nightly. Bupropion XL was increased to 300 mg in the morning, while fluoxetine 60 mg and risperidone 1 mg were continued. This split-dosing approach is consistent with preliminary case-based observations indicating that some patients experience diurnal symptom recurrence when dextromethorphan and piracetam are administered only nightly [18].

On Day 83, the patient reported a similar level of frequent obsessions, but she could use a clearer mind to overcome them more often when struggling. Some hypochondriacal thoughts had appeared. Mood was not too low. Bathing time had decreased to approximately 30 minutes. Buying impulses persisted, including the purchase of many dolls. Sweating was prominent. Fluoxetine was reduced to 40 mg, grounding techniques were taught, and dextromethorphan, bupropion, piracetam, and risperidone were continued.

On Day 111, PHQ-9 was 11, and GAD-7 was 10. Passive suicidal ideation was again endorsed for several days on the PHQ-9. Still, the clinical notes emphasized fewer spells of low mood and better-than-expected emotional regulation in the context of her elderly dog’s illness. She reported some nightmares with sweating, hand sweating in the morning, and improved ability to resist obsessive-compulsive thoughts for longer periods. Propranolol 20 mg twice daily was planned for somatic anxiety and sweating, fluoxetine was reduced to 30 mg, and risperidone was increased to 1.5 mg.

On Day 125, her dog had passed away. The patient’s condition had worsened quickly and dramatically in response to the loss, but her emotional reaction was still better than she had expected. She attended the funeral and did not overreact. Hand sweating had reduced overall, though nightmares continued. Obsession frequency and obsessive-compulsive symptoms remained similar. Clomipramine 25 mg nightly was added, and fluoxetine was reduced to 20 mg. Senna was prescribed to manage the anticipated constipation risk. The rest of the regimen included bupropion XL 300 mg in the morning, dextromethorphan 45 mg twice daily, piracetam 600 mg twice daily, propranolol 20 mg twice daily, and risperidone 1.5 mg nightly.

By Day 140, clomipramine was increased to 50 mg nightly, fluoxetine was reduced to 10 mg daily, and citicoline 500 mg was added in the morning. The glutamatergic backbone remained dextromethorphan 45 mg twice daily and piracetam 600 mg twice daily, with bupropion XL 300 mg in the morning, propranolol, and risperidone 1.5 mg nightly.

On Day 169, the patient reported constipation after adding clomipramine and amenorrhea after risperidone. Her mood was stable, but she remained anxious about touching dirty objects. The clinician explicitly reminded her that she could not depend solely on medication and should sometimes resist compulsions. Bisacodyl was added for constipation. The pharmacological regimen was otherwise continued.

On Day 197, L-glutamine 500 mg daily was introduced, and risperidone was reduced to 0.5 mg nightly. The continued regimen included bupropion XL 300 mg, dextromethorphan 45 mg twice daily, piracetam 600 mg twice daily, clomipramine 50 mg nightly, and L-glutamine. The addition of L-glutamine was consistent with the broader CGR rationale of supporting glutamate-glutamine cycling and glutamatergic substrate availability [14,22,23].

On Day 225, the patient reported less tiredness after reducing risperidone. She had completed two short periods of promoter work, attended marketing or public relations interviews, maintained exercise, and described motivation as acceptable despite uncertainty and unsuccessful interviews. She had also learned about color analysis in Korea and studied online to strengthen her marketing materials and related concepts. Her parents reportedly scolded her less because she was actively seeking jobs. The regimen continued, and trihexyphenidyl was added, although the notes did not specify the exact target symptom.

On Day 253, menstruation had become more regular following a reduction in risperidone. She still felt somewhat tired on waking, reported occasional shortness-of-breath sensations, dreams affecting sleep quality, bruising on and off, possible hemorrhoids, and possible clomipramine-related issues. Low mood and fragmented sleep were noted. Paroxetine controlled-release 6.25 mg nightly was added, pregabalin 50 mg nightly was reintroduced, and cyproheptadine 4 mg twice daily was added to reduce sweating. Clomipramine was no longer listed in the medication section at this visit, suggesting it had been stopped or withheld in the context of tolerability concerns. However, the note only stated “to reduce” and did not specify the final clomipramine decision. Therefore, the final clomipramine disposition should be regarded as unclear from the available record. The regimen at this point included bupropion XL 300 mg, dextromethorphan 45 mg twice daily, piracetam 600 mg twice daily, risperidone 0.5 mg nightly, L-glutamine 500 mg daily, paroxetine controlled-release 6.25 mg nightly, pregabalin 50 mg nightly, and cyproheptadine.

Adverse effects and management

Adverse effects were managed by dose adjustment rather than broad discontinuation. Fatigue was prominent early and again later, when risperidone levels were higher. Reducing risperidone from 1.5 mg to 0.5 mg was followed by less tiredness and more regular menstruation, suggesting that antipsychotic-related sedation and prolactin-related menstrual effects were clinically relevant. This is consistent with known tolerability concerns in antipsychotic augmentation, including sedation, metabolic issues, and endocrine effects [4,5,24].

Sweating emerged during serotonergic and dextromethorphan-based treatments and was managed with propranolol and, later, cyproheptadine. Given that dextromethorphan has serotonergic properties and was combined with CYP2D6 inhibitors, monitoring for serotonin toxicity was important [19,20,25]. No full serotonin syndrome was recorded in this case. Constipation appeared after clomipramine was introduced and was managed with senna and bisacodyl. Pregabalin was useful for nightmares and later reintroduced for fragmented sleep.

Patient-level safety documentation was limited by the retrospective routine-care nature of the report. A prior ECG obtained for chest symptoms was reportedly unremarkable. Still, the available notes did not document post-escalation ECG monitoring, serial vital-sign tables, a structured neurological checklist for clonus or hyperreflexia, prolactin measurement after amenorrhea, pregnancy testing, or laboratory assessment for bruising/bleeding complaints. The record also did not document fever, clonus, confusion, severe autonomic instability, mania, psychosis, or dissociative reaction. These gaps limit safety interpretation and should not be taken as evidence that unrecorded assessments were normal or abnormal.

Concomitant interventions

The record documented previous counseling and psychological input. During pharmacological treatment, grounding techniques were taught, and the patient was encouraged to resist compulsions at times. The notes specifically emphasized that medication alone would not be sufficient for residual contamination fears. No formal exposure and response prevention protocol, home exposure hierarchy, or Yale-Brown Obsessive Compulsive Scale measurement was recorded. Medication adherence was assessed clinically through follow-up discussions and prescription adjustments; no pill counts, pharmacy refill analyses, or formal adherence scales were recorded.

Clinical course and outcome

Across the observation period, the patient showed a mixed but clinically meaningful response. The clearest quantitative improvement was in depressive and anxiety screening early in treatment. PHQ-9 decreased from 18 on Day 16 to 10 on Day 41, while GAD-7 decreased from 14 to 9 over the same interval. The PHQ-9 suicidal ideation item changed from “several days” to “not at all” by Day 41. On Day 111, PHQ-9 was 11, and GAD-7 was 10 during the period of her dog’s serious illness, and passive suicidal ideation was again endorsed for several days. Clinical notes nevertheless described fewer low mood spells and better-than-expected emotional control. Later notes did not provide further PHQ-9 or GAD-7 values. Thus, the strongest standardized data concerned depression and anxiety, while suicidal ideation showed fluctuation under stress rather than a simple linear resolution.

Obsessive-compulsive improvement was partial. On Day 27, the patient described rituals as only slightly improved, by approximately 20-30%. On Day 41, bathing time had slightly decreased, and by Day 83, it had decreased from the initial 1.5 hours to approximately 30 minutes, a meaningful functional change. However, fears of contamination, repeated handwashing, and concerns about home-centered cleaning persisted. The core residual cluster remained most prominent at home. She repeatedly reported that she could function better outside than at home, suggesting a strong context-specific expression of symptoms. Because no Y-BOCS, structured ritual count, contamination hierarchy, or home-versus-outside severity rating was recorded, OCD improvement should be interpreted mainly from charted clinical estimates and bathing duration rather than from standardized OCD-specific measurement.

Several non-obsessive-compulsive domains improved more robustly. Chest dullness decreased early and was later absent. Mood became more stable with less fluctuation. She became more relaxed and more able to pack her belongings. Motivation improved over time. After risperidone was reduced and the glutamatergic backbone was maintained, she completed short-term promotional work, attended marketing and public relations interviews, continued exercising, learned about color analysis, and studied online to improve her marketing materials. These functional changes were clinically important because her last full-time job had ended approximately two years earlier, and paternal criticism about underemployment had been a major stressor.

The response to pet loss was also notable. When her elderly dog became ill and later died, the patient’s condition worsened quickly, but she reported that her emotions were better than expected. She attended the funeral and did not overreact. This did not represent the absence of grief. Rather, it suggested improved emotional containment and resilience compared with what she and the clinician anticipated. This kind of functional resilience is not fully captured by symptom scales, but it mattered to the patient’s recovery.

Side effects improved with personalization. Fatigue became less impactful over time and improved after a reduction in risperidone. Amenorrhea improved after reducing risperidone. Sweating was addressed with propranolol and later cyproheptadine. Constipation after clomipramine was monitored and treated. Pregabalin reduced the distressing effect of nightmares and was later reintroduced when sleep became fragmented. No psychotic, manic, or dissociative reaction was recorded.

In summary, the outcome was not remission of contamination-focused OCD. The better description is incremental recovery: reduced bathing time, improved mood and energy, reduced somatic chest symptoms, greater motivation, better work-seeking behavior, improved ability to resist obsessive-compulsive thoughts for longer periods, and better coping with a major attachment loss. Persistent home-based contamination rituals remained the main unresolved problem. The improvement occurred during a complex period of overlapping medication changes and cannot be attributed confidently to any single medication, medication class, or proposed mechanism.

Patient perspective

The patient’s perspective was available through clinical notes rather than through a separate written statement. She reported feeling less tired, more relaxed, and at times clearer in resisting obsessive thoughts. She remained distressed by home-based contamination rituals, but she valued being able to attend interviews, exercise, learn new skills, and cope better than expected with her dog’s illness and death. No separate verbatim patient-perspective form was obtained.

## Discussion

Interpretation of partial obsessive-compulsive improvement and broader functional gains

This case illustrates a pattern that is common in difficult OCD but often underreported: core rituals may remain while the patient becomes more functional, less demoralized, and more able to resist or delay symptoms. The patient did not achieve full remission. She continued to experience contamination fears, repeated handwashing, and home-dominant cleaning concerns. Yet several changes were clinically meaningful. Bathing time decreased from approximately 1.5 hours to 30 minutes. The PHQ-9 self-harm item improved by Day 41 but later fluctuated during stress, emphasizing the need for longitudinal rather than single-time-point interpretation. Chest dullness improved, mood became more stable, and she resumed job-seeking, exercise, and skill-building.

This distinction matters. In refractory OCD, measuring only ritual frequency may miss important gains in energy, resilience, self-efficacy, and daily functioning. Conversely, improved mood should not be mistaken for the resolution of OCD. In this case, affective and motivational gains appeared stronger than the reduction in core home-based contamination symptoms. That pattern supports a layered formulation: the regimen may have improved mood, fatigue, somatic distress, and cognitive flexibility, while deeply reinforced home rituals required additional behavioral and family-context intervention. However, because treatment involved sequential and overlapping medication changes, this interpretation remains clinical and hypothesis-generating rather than causal.

Home-dominant symptoms and contextual learning

**Figure 1:**
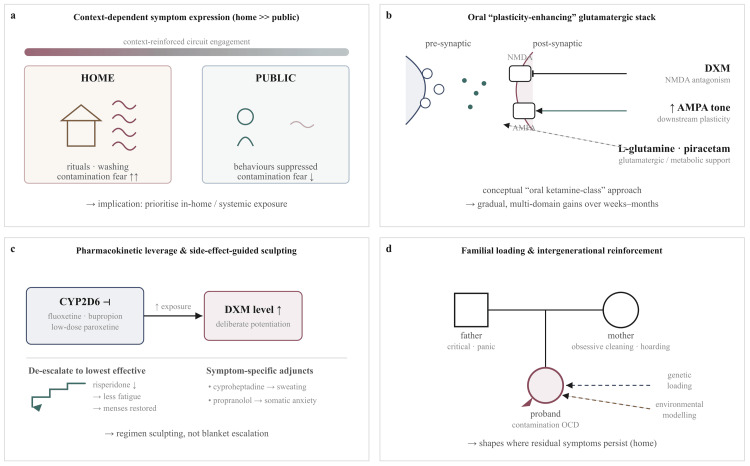
Schematic synthesis of four high-yield clinical insights presented as mechanism and reasoning concepts rather than empirical results (a) Context-dependent expression of obsessive-compulsive symptoms, markedly intensified in the home relative to public settings, implicating context-reinforced circuit engagement and favoring in-home/systemic exposure work. (b) Rationale for an oral, plasticity-oriented glutamatergic stack combining NMDA antagonism (dextromethorphan, DXM), enhanced AMPA-mediated downstream plasticity, and glutamatergic/metabolic support (L-glutamine, piracetam). (c) Deliberate pharmacokinetic leverage, CYP2D6 inhibition (fluoxetine, bupropion, low-dose paroxetine) to raise DXM exposure, paired with side-effect-guided de-escalation (antipsychotic minimization) and symptom-specific adjuncts. (d) Intergenerational familial loading (maternal obsessive cleaning/hoarding; paternal critical/panic traits), combining probable genetic enrichment with environmental modeling. This figure is conceptual and should not be read as proof of a mechanism in this patient. DXM: dextromethorphan, NMDA: N-methyl-D-aspartate, AMPA: α-amino-3-hydroxy-5-methyl-4-isoxazolepropionic acid, OCD: obsessive-compulsive disorder Image credit: N Cheung using PowerPoint (Microsoft Corp., Redmond, WA, USA).

One of the most important clinical observations was that obsessive-compulsive symptoms were far worse at home than outside (Figure 1). The patient could go out, attend interviews, work in limited roles, and pursue learning activities, yet contamination concerns and cleaning rituals persisted at home. This home-public dissociation may have several explanations. Home is the place where washing materials are available, privacy allows rituals to expand, and family interactions may trigger shame or doubt. The patient’s mother’s long-standing cleaning behavior and detergent hoarding may also have modeled or normalized contamination vigilance. Her father’s criticism may have increased threat sensitivity and self-doubt, indirectly strengthening compulsive control behaviors.

Family accommodation and home reinforcement are well-described in OCD [6]. Avoidance is also central to obsessive-compulsive phenomenology and can become context-dependent [7]. In this case, the persistence of home-based rituals despite broader functional improvement suggests that medication alone was unlikely to extinguish the learned home ritual network. A home-targeted exposure and response prevention plan would likely be necessary, ideally with careful attention to family accommodation, criticism, and the patient’s fear of invalidation.

Glutamatergic and plasticity-based rationale

The pharmacological strategy was centered on the clinical hypothesis that glutamatergic modulation could improve plasticity in refractory symptoms. Dextromethorphan was used as an NMDA/sigma-related agent. Fluoxetine, bupropion, and later low-dose paroxetine were used not only as antidepressant agents but also as CYP2D6 inhibitors that could prolong dextromethorphan exposure. Piracetam was used as an AMPA-related modulator, and L-glutamine was later added to support glutamate-glutamine cycling.

This approach is consistent with the broader CGR proposal [14]. It also aligns with the glutamatergic model of OCD described by Pittenger et al. [9] and with cortico-striatal models emphasizing circuit-level dysregulation [8]. Ketamine’s rapid effects are thought to involve changes in NMDA and AMPA signaling, mTOR-related synapse formation, and downstream plasticity [11-13]. Piracetam has been reported to interact with AMPA receptor modulation and has a long history as a nootropic agent, though its psychiatric evidence base remains limited [26,27]. Glutamine has been studied in relation to glutamatergic activity and stress-related cognitive effects, including preclinical evidence that supplementation may reduce chronic stress-associated cognitive impairment and oxidative stress [22,23].

In this patient, the most plausible interpretation is not that the regimen directly “cured” contamination-focused OCD. Rather, the regimen may have improved the patient’s capacity to resist, recover, and function while partially reducing obsessive-compulsive intensity. The note that “her clear mind can win over in more times” captures this well. The change was not simply fewer obsessions; it was a modest shift in the balance between obsessional pressure and cognitive control. Still, no glutamate biomarker, pharmacokinetic level, neuroimaging measure, or experimental challenge was obtained, so the proposed glutamatergic and plasticity mechanisms were not directly demonstrated in this case.

Comparison with conventional augmentation

Standard care for refractory OCD often involves high-dose serotonergic medication, clomipramine, antipsychotic augmentation, and exposure-based psychotherapy [1,2]. The patient received several components of this approach, including fluoxetine, risperidone, and, later, clomipramine. Risperidone augmentation has evidence but is limited by tolerability and may be inferior to exposure and response prevention augmentation for some patients [5]. In this case, increasing risperidone did not resolve core obsessive-compulsive symptoms and likely contributed to fatigue and menstrual disturbance. Reducing risperidone improved tiredness and menstrual regularity, supporting the importance of using the lowest helpful antipsychotic dose.

Clomipramine was added when obsessive-compulsive symptoms remained persistent, but constipation and possible tolerability issues emerged. This is clinically unsurprising given clomipramine’s anticholinergic burden. The latter regimen moved toward lower risperidone exposure, continued dextromethorphan-piracetam-glutamine support, and symptom-targeted adjuncts. This pattern aligns with the practical optimization approach described in CGR clinical reports, in which medication selection is adjusted based on response, side effects, and pharmacokinetic considerations rather than following a fixed protocol [17,18]. Because these optimization reports are preliminary and largely case-based, they should be interpreted as clinical observations rather than as established guidance.

Pharmacokinetic personalization and safety

**Figure 2:**
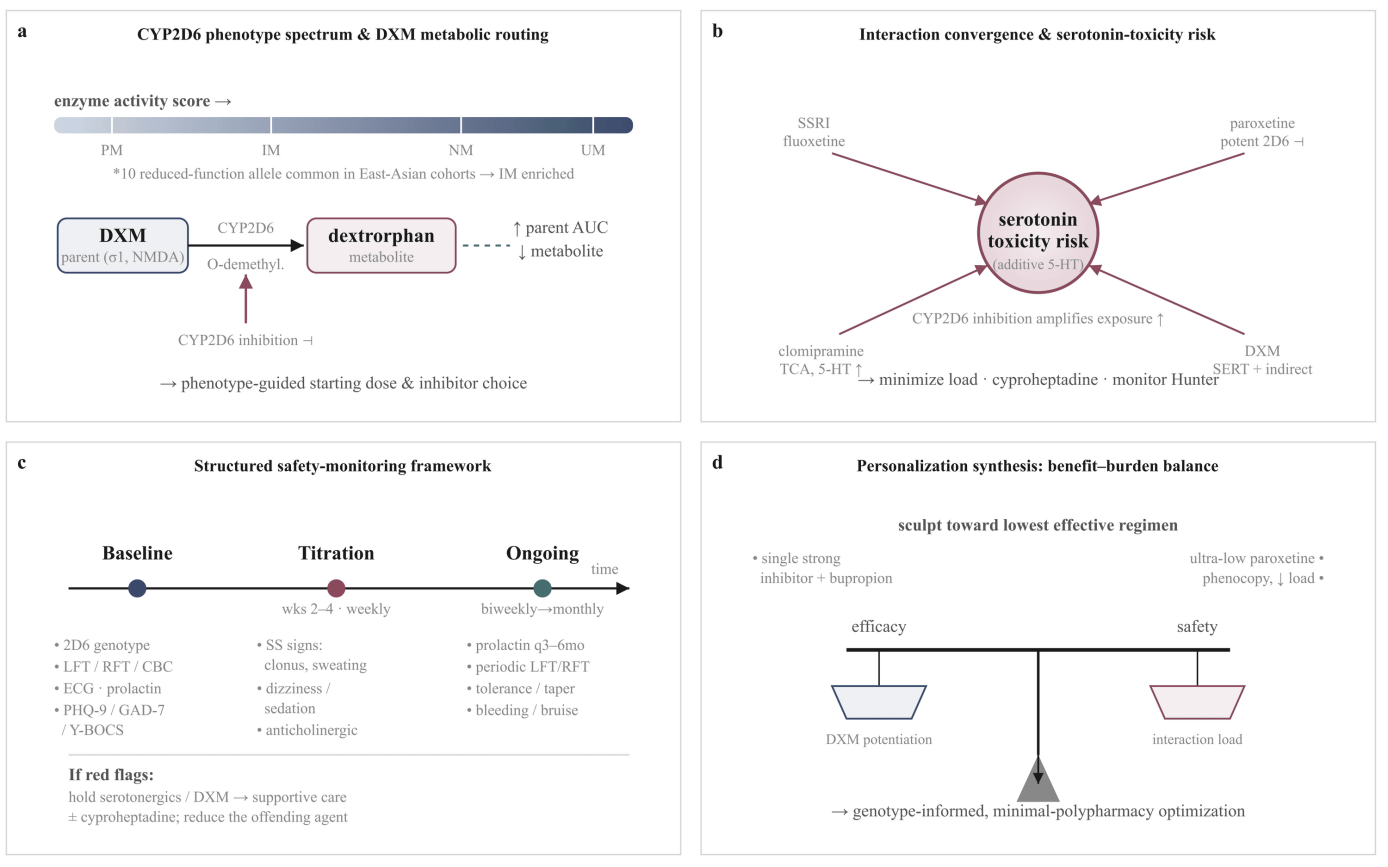
Conceptual schematic of the pharmacokinetic-personalization and safety strategy (a) The CYP2D6 activity-score spectrum (poor to ultrarapid metabolizer; intermediate-metabolizer enrichment with the reduced-function *10 allele in East Asian cohorts) and the metabolic routing of dextromethorphan (DXM) to dextrorphan, with CYP2D6 inhibition deliberately raising parent-drug exposure to inform genotype/phenotype-guided dosing. (b) Convergence of multiple serotonergic agents (SSRI, paroxetine, clomipramine, and DXM) onto an additive serotonin-toxicity risk node, amplified by CYP2D6 inhibition, with mitigation by load reduction, cyproheptadine, and structured surveillance. (c) A phased monitoring framework spanning baseline work-up, intensive titration-phase surveillance for serotonin syndrome and anticholinergic signs, and longer-term laboratory follow-up, with predefined escalation rules. (d) The personalization principle as a benefit-burden balance, sculpting toward the lowest effective regimen via a single strong inhibitor plus bupropion or ultra-low paroxetine phenocopy. This figure represents a proposed safety framework and was not fully documented as implemented in this patient. DXM: dextromethorphan, CYP2D6: cytochrome P450 2D6, PM/IM/NM/UM: poor/intermediate/normal/ultrarapid metabolizer, SSRI: selective serotonin reuptake inhibitor, TCA: tricyclic antidepressant, SS: serotonin syndrome, Y-BOCS: Yale-Brown Obsessive Compulsive Scale Image credit: N Cheung using PowerPoint (Microsoft Corp., Redmond, WA, USA).

The case also highlights the double-edged nature of CYP2D6 inhibition (Figure 2). Fluoxetine and paroxetine inhibit CYP2D6, and bupropion can produce clinically meaningful CYP2D6 inhibition through both reversible inhibition and downregulation [19,21]. This can be useful when the aim is to prolong dextromethorphan exposure, but it also raises the risk of excessive serotonergic or neuropsychiatric effects. Dextromethorphan-bupropion is an approved antidepressant combination in some settings, but the broader combination of dextromethorphan with SSRIs, clomipramine, or multiple CYP2D6 inhibitors requires caution [20]. Serotonin syndrome has been reported with dextromethorphan and serotonergic antidepressants [25].

In this patient, sweating was noted and attributed by the patient to “serotonin” on one side effect form. The record did not document fever, clonus, confusion, severe autonomic instability, or a diagnosis of serotonin syndrome. Still, the emergence of sweating led to dose reduction and symptom-specific management. This is important because early autonomic symptoms can be mistaken for anxiety. The late addition of low-dose paroxetine may have further inhibited CYP2D6, but it also increased the need for careful monitoring. The available record did not include dextromethorphan levels, CYP2D6 genotype, or a structured serotonin toxicity checklist, which limits the interpretation of pharmacokinetic personalization.

Split dosing was another personalization step. When dextromethorphan and piracetam were expanded to morning and evening dosing, the dosing schedule resembled recent case-based observations, suggesting that twice-daily dosing may help patients with diurnal symptom recurrence [18]. In this patient, the record does not clearly state a late-afternoon wearing-off pattern. However, split dosing was used as part of the optimization strategy and coincided with later gains in bathing time and functioning. This temporal association should not be read as evidence that split dosing caused the improvement.

Somatic and hypochondriacal features

The patient’s symptoms included chest dullness, dizziness, sweating, irritable bowel symptoms, and later transient hypochondriacal thoughts. These symptoms did not appear separate from the obsessive-compulsive and anxiety presentation. Instead, they formed a somatic-interoceptive layer of the illness. Somatic symptom disorder, health anxiety, and OCD can overlap, especially when bodily sensations become the focus of repetitive checking, worry, or reassurance seeking [28]. Cheung’s case-oriented reports on somatic and hypochondriacal obsessions provide a related clinical precedent for glutamatergic augmentation in such presentations, although those reports remain preliminary [29,30].

In this case, chest dullness improved early and later disappeared, even while contamination fears persisted. This suggests that somatic anxiety may have been more responsive than the home-based cleaning circuit. That difference may help guide treatment: somatic distress and affective instability may improve pharmacologically, while cleaning rituals may require more deliberate exposure-based retraining. Again, this is a clinical interpretation rather than a demonstrated separation of mechanisms.

Nightmares, sleep, and subthreshold trauma features

Nightmares were present from the first visit and sometimes involved people who had hurt or traumatized the patient in the past. She also woke with shortness of breath. These symptoms do not establish posttraumatic stress disorder from the available record, but they suggest trauma-related sleep disturbance or subthreshold trauma features. Pregabalin appeared to reduce nightmare recall or distress early and was later reintroduced when sleep became fragmented. Propranolol and cyproheptadine were used for sweating and autonomic symptoms. The record, therefore, supports treating sleep and autonomic arousal as separable targets rather than assuming they will resolve automatically when obsessive-compulsive symptoms improve.

The patient’s response to the illness and death of her elderly dog was clinically meaningful. She worsened quickly, but she coped better than expected, attended the funeral, and did not overreact. This may reflect improved resilience rather than symptom remission. In real-world care, such resilience markers are often as important as scale scores because they predict whether a patient can continue to function under stress.

Familial loading and intergenerational patterns

The patient’s family history suggested both genetic and environmental loading. Her mother had prominent cleaning and detergent-hoarding behaviors, and her father’s criticism and possible panic symptoms contributed to a stressful home environment. The patient’s symptoms being worse at home than outside may partly reflect this intergenerational and relational context. Recent mechanistic writing has proposed that OCD may involve synaptic pruning and vulnerability to plasticity, although these models remain exploratory and require independent validation [14]. Whether such models ultimately prove correct or not, the clinical point is straightforward: the family environment likely shaped symptom expression and should be included in treatment planning. No family member was formally assessed, and no diagnosis should be inferred in relatives from these contextual observations alone.

Limitations

This report has several limitations. It is a single case from routine practice and cannot establish causality. Multiple medications were changed over time, including fluoxetine, bupropion, dextromethorphan, risperidone, piracetam, clomipramine, propranolol, pregabalin, L-glutamine, paroxetine, and cyproheptadine. Improvements cannot be attributed to one agent. This confounding due to polypharmacy is a major limitation because the glutamatergic-oriented strategy was embedded within an evolving serotonergic, antipsychotic, anxiolytic, sleep-focused, and symptom-directed regimen.

Formal obsessive-compulsive severity ratings such as the Yale-Brown Obsessive Compulsive Scale were not recorded, and ritual frequency was not systematically logged. Exposure and response prevention was not delivered in a structured, measured way, and family accommodation was not formally assessed. Therefore, the OCD-specific outcome evidence is weaker than the PHQ-9/GAD-7 data and the bathing-time estimate. The case also involved side effects and pharmacokinetic complexity, making the regimen unsuitable for unsupervised use.

Safety reporting was also incomplete. The available notes did not provide serial vital signs, ECG monitoring after medication escalation, prolactin measurement after amenorrhea, pregnancy testing, laboratory assessment for bruising or bleeding, pharmacokinetic levels, or structured serotonin-syndrome monitoring. Adherence was not measured with pill counts or a formal adherence instrument. The patient's perspective was reconstructed from clinic notes rather than obtained as a separate written statement. Finally, several supporting reports for the proposed regimen are preliminary preprints or case-based publications from the same authorial group, and independent replication is lacking.

Future directions

Future studies should test oral glutamatergic augmentation in larger samples with standardized obsessive-compulsive measures, adverse-event monitoring, and clear medication protocols. For contamination-focused OCD, future work should include home-specific outcome measures because clinic-based improvement may not reflect the most impaired environment. Combining glutamatergic augmentation with home-targeted exposure and response prevention may be especially important. Biomarkers related to glutamate, synaptic plasticity, sleep, fatigue, and interoceptive symptoms may also help identify which patients are most likely to benefit. Future reports should also include clearer active-regimen tables, start-stop medication dates, adherence assessment, structured tolerability monitoring, and patient-reported outcomes.

## Conclusions

This case describes a 26-year-old woman with refractory, home-dominant contamination-focused OCD complicated by anxiety, depression, somatic symptoms, nightmares, fatigue, and motivational impairment. During a complex outpatient treatment course, a personalized oral glutamatergic regimen centered on dextromethorphan, piracetam, and L-glutamine, with CYP2D6-inhibiting antidepressants and carefully adjusted adjuncts, was associated with meaningful but incomplete improvement. Bathing time fell from approximately 1.5 hours to 30 minutes; mood and energy improved; somatic chest symptoms decreased; and functional motivation returned through job-seeking, exercise, and skill acquisition. The PHQ-9 self-harm item improved at Day 41 but later fluctuated during stress, and no later standardized suicidality rating was recorded.

The core residual problem remained home-based contamination and cleaning rituals, emphasizing that medication alone was insufficient. Because there was no Y-BOCS, no structured ritual count, no measured home-versus-outside scale, and substantial confounding due to polypharmacy, the case cannot establish the efficacy of the glutamatergic regimen or any individual component. The case raises a hypothesis for further controlled study of dextromethorphan-centered glutamatergic-oriented augmentation in refractory obsessive-compulsive presentations, while also highlighting the need for careful pharmacokinetic monitoring and integration with home-focused exposure-based therapy.

## References

[1] Hirschtritt ME, Bloch MH, Mathews CA (2017). Obsessive-compulsive disorder: advances in diagnosis and treatment. JAMA.

[2] Stein DJ, Costa DL, Lochner C (2019). Obsessive-compulsive disorder. Nat Rev Dis Primers.

[3] Bloch MH, McGuire J, Landeros-Weisenberger A, Leckman JF, Pittenger C (2010). Meta-analysis of the dose-response relationship of SSRI in obsessive-compulsive disorder. Mol Psychiatry.

[4] Dold M, Aigner M, Lanzenberger R, Kasper S (2015). Antipsychotic augmentation of serotonin reuptake inhibitors in treatment-resistant obsessive-compulsive disorder: an update meta-analysis of double-blind, randomized, placebo-controlled trials. Int J Neuropsychopharmacol.

[5] Simpson HB, Foa EB, Liebowitz MR (2013). Cognitive-behavioral therapy vs risperidone for augmenting serotonin reuptake inhibitors in obsessive-compulsive disorder: a randomized clinical trial. JAMA Psychiatry.

[6] Lebowitz ER, Panza KE, Su J, Bloch MH (2012). Family accommodation in obsessive-compulsive disorder. Expert Rev Neurother.

[7] McGuire JF, Storch EA, Lewin AB, Price LH, Rasmussen SA, Goodman WK (2012). The role of avoidance in the phenomenology of obsessive-compulsive disorder. Compr Psychiatry.

[8] Milad MR, Rauch SL (2012). Obsessive-compulsive disorder: beyond segregated cortico-striatal pathways. Trends Cogn Sci.

[9] Pittenger C, Bloch MH, Williams K (2011). Glutamate abnormalities in obsessive compulsive disorder: neurobiology, pathophysiology, and treatment. Pharmacol Ther.

[10] Rodriguez CI, Kegeles LS, Levinson A (2013). Randomized controlled crossover trial of ketamine in obsessive-compulsive disorder: proof-of-concept. Neuropsychopharmacology.

[11] Koike H, Iijima M, Chaki S (2011). Involvement of AMPA receptor in both the rapid and sustained antidepressant-like effects of ketamine in animal models of depression. Behav Brain Res.

[12] Li N, Lee B, Liu RJ (2010). mTOR-dependent synapse formation underlies the rapid antidepressant effects of NMDA antagonists. Science.

[13] Zanos P, Moaddel R, Morris PJ (2016). NMDAR inhibition-independent antidepressant actions of ketamine metabolites. Nature.

[14] Cheung N (2026). DXM, CYP2D6-inhibiting antidepressants, piracetam, and glutamine: proposing a ketamine-class antidepressant regimen with existing drugs. Front Psychiatry.

[15] Cheung N (2025). Case series: marked improvement in treatment-resistant obsessive-compulsive symptoms with over-the-counter glutamatergic augmentation in routine clinical practice (Preprint). Preprints.org.

[16] Cheung N (2025). Cheung’s regimen series: successful conversion from one dose of esketamine to a low-cost oral ketamine-class glutamatergic regimen in treatment-resistant depression and OCD (Preprint). Preprints.org.

[17] Cheung N (2025). Clinical experience and optimisation of the Cheung glutamatergic regimen for refractory psychiatric diseases (Preprint). Preprints.org.

[18] Cheung N (2025). Dosing schedules for dextromethorphan and piracetam in OCD: a case series on diurnal symptom patterns and split-dosing strategies (Preprint). Preprints.org.

[19] Crewe HK, Lennard MS, Tucker GT, Woods FR, Haddock RE (1992). The effect of selective serotonin re-uptake inhibitors on cytochrome P4502D6 (CYP2D6) activity in human liver microsomes. Br J Clin Pharmacol.

[20] McCarthy B, Bunn H, Santalucia M, Wilmouth C, Muzyk A, Smith CM (2023). Dextromethorphan-bupropion (Auvelity) for the treatment of major depressive disorder. Clin Psychopharmacol Neurosci.

[21] Sager JE, Tripathy S, Price LS, Nath A, Chang J, Stephenson-Famy A, Isoherranen N (2017). In vitro to in vivo extrapolation of the complex drug-drug interaction of bupropion and its metabolites with CYP2D6; simultaneous reversible inhibition and CYP2D6 downregulation. Biochem Pharmacol.

[22] Baek JH, Jung S, Son H, Kang JS, Kim HJ (2020). Glutamine supplementation prevents chronic stress-induced mild cognitive impairment. Nutrients.

[23] Son H, Baek JH, Go BS (2018). Glutamine has antidepressive effects through increments of glutamate and glutamine levels and glutamatergic activity in the medial prefrontal cortex. Neuropharmacology.

[24] Reynolds GP, Kirk SL (2010). Metabolic side effects of antipsychotic drug treatment--pharmacological mechanisms. Pharmacol Ther.

[25] Dy P, Arcega V, Ghali W, Wolfe W (2017). Serotonin syndrome caused by drug to drug interaction between escitalopram and dextromethorphan. BMJ Case Rep.

[26] Ahmed AH, Oswald RE (2010). Piracetam defines a new binding site for allosteric modulators of alpha-amino-3-hydroxy-5-methyl-4-isoxazole-propionic acid (AMPA) receptors. J Med Chem.

[27] Winblad B (2005). Piracetam: a review of pharmacological properties and clinical uses. CNS Drug Rev.

[28] Henningsen P (2018). Management of somatic symptom disorder. Dialogues Clin Neurosci.

[29] Cheung N (2025). OTC glutamatergic augmentation resolves adolescent refractory somatic symptoms (Preprint). Preprints.org.

[30] Cheung N (2025). Rapid remission of refractory hypochondriacal OCD in an elderly patient under glutamatergic augmentation: a high-resolution case observation (Preprint). Preprints.org.

